# Remote-sensing based approach to forecast habitat quality under climate change scenarios

**DOI:** 10.1371/journal.pone.0172107

**Published:** 2017-03-03

**Authors:** Juan M. Requena-Mullor, Enrique López, Antonio J. Castro, Domingo Alcaraz-Segura, Hermelindo Castro, Andrés Reyes, Javier Cabello

**Affiliations:** 1 Andalusian Center for the Assessment and Monitoring of Global Change (CAESCG), University of Almería, Almería, Spain; 2 Didactics of Experimental Sciences Area, Department of Education, University of Almería, La Cañada de San Urbano, Almería, Spain; 3 Department of Biological Sciences, Idaho State University, Gale Life Sciences Bldg. Rm 207, 8th Avenue, Mail Stop, Pocatello, ID, United States of America; 4 Department of Botany, University of Granada, Granada, Spain; 5 Department of Biology and Geology, University of Almería, La Cañada de San Urbano, Almería, Spain; University of Vigo, SPAIN

## Abstract

As climate change is expected to have a significant impact on species distributions, there is an urgent challenge to provide reliable information to guide conservation biodiversity policies. In addressing this challenge, we propose a remote sensing-based approach to forecast the future habitat quality for European badger, a species not abundant and at risk of local extinction in the arid environments of southeastern Spain, by incorporating environmental variables related with the ecosystem functioning and correlated with climate and land use. Using ensemble prediction methods, we designed global spatial distribution models for the distribution range of badger using presence-only data and climate variables. Then, we constructed regional models for an arid region in the southeast Spain using EVI (Enhanced Vegetation Index) derived variables and weighting the pseudo-absences with the global model projections applied to this region. Finally, we forecast the badger potential spatial distribution in the time period 2071–2099 based on IPCC scenarios incorporating the uncertainty derived from the predicted values of EVI-derived variables. By including remotely sensed descriptors of the temporal dynamics and spatial patterns of ecosystem functioning into spatial distribution models, results suggest that future forecast is less favorable for European badgers than not including them. In addition, change in spatial pattern of habitat suitability may become higher than when forecasts are based just on climate variables. Since the validity of future forecast only based on climate variables is currently questioned, conservation policies supported by such information could have a biased vision and overestimate or underestimate the potential changes in species distribution derived from climate change. The incorporation of ecosystem functional attributes derived from remote sensing in the modeling of future forecast may contribute to the improvement of the detection of ecological responses under climate change scenarios.

## Introduction

As climate change is expected to have a significant impact on species distributions, there is an urgent challenge to provide policy-makers with valuable information to guide conservation biodiversity policies. To address this challenge, modeling approaches should be enhanced with the aim of increasing the confidence of the future forecasting [1]. In particular, mammalian species richness will be dramatically reduced throughout the Mediterranean basin; however, the trend will not be uniform for all taxa. For instance, Mustelidae (e.g., badger, weasel) will decrease while Canidae (e.g., wolf), Hyaenidae (e.g., hyena) and some families of Chiroptera (bats) will increase [2]. These findings highlight the complexity of species response to climate change and the necessity of focusing modeling efforts to enhance the reliability of the predicted information.

Species distribution models (SDMs) are used to guide conservation programs in anticipation of future climate change effects on species [3]. SDMs capture relationships between a species (occurrence) and its environment. However, some authors recognize that the incomplete coverage of the environmental niche of species is one of the important sources of error when forecasting distributions under climate change, because it does not cover completely the spatial distribution range of species [4], and/or only consider climatic variables as predictors [5]. In addition, forecasts of species distribution under climate change entail an inherent uncertainty such as residual error of models, modeling algorithm and climate scenarios selected [6]. Different solutions have been proposed for solving these issues. For example, Gallien et al. [7] provide a methodological framework where the use of predictions based on a global model to weight pseudo-absences in a regional model significantly improved the predictive performance of regional SDMs. Wenger et al. [6] suggested a Monte Carlo approach that accounts for uncertainty within generalized linear regression models. Finally, the incorporation of other environmental variables (different than climate ones), e.g., land use or ecosystem functioning descriptors such as ecosystem production and seasonality, would improve the reliability of the predictions [5,8]. However, we suggest that it is also necessary to incorporate the uncertainty derived of the future values of projected environmental variables. With this aim, we have developed a remote-sensing based approach.

Remotely sensed indicators of ecosystem functioning are increasingly being used in animal research. In particular, spectral Vegetation Indices (VIs) have been used to great success in mammal ecology [9]. VIs are conceptually and empirically linked with primary production [10], which determines the amount of green biomass available for herbivores and is referred as the main descriptor of ecosystem functioning [11].

Functional attributes derived from VIs are usually expressed as average temporal summaries, such as the annual mean (i.e., surrogate of mean annual primary production) or the seasonal coefficient of variation (i.e., indicator of seasonality or temporal variation within the year) [12]. Of particular note are spectral Vegetation Indices (VIs), such as the Normalized Difference Vegetation Index (NDVI) and the Enhanced Vegetation Index (EVI). Both of these VIs are directly related with the fraction of photosynthetically active radiation (fAPAR) intercepted by green vegetation [13]. This relationship allows the derivation of regional maps of primary production from radiation use efficiency values [14]. Landscape functional heterogeneity has also been suggested as a significant driver of species [15] and ecosystem diversity [12], particularly in the Mediterranean Region. Many animal species have proved to be especially sensitive to spatial heterogeneity [16]. Recent findings suggest that this sensitivity is related more to functional heterogeneity than to structural heterogeneity [17]. For instance, Requena-Mullor et al. [8] found modeled spatial distribution of the European badger (*Meles meles* L.) in SE Spain was significantly improved when augmenting climate variables with EVI-derived functional attributes, (esp. the spatial variability of EVI) rather than land cover and land use variables.

The purpose of this study is to explore the benefits of including remotely sensed descriptors of the temporal dynamics and spatial patterns of ecosystem functioning into spatial distribution models in order to enhance the reliability of future forecasts of terrestrial mammal habitat quality. We compared the future forecasts obtained by using models with and without incorporating remotely sensed descriptors. We used the European badger in an arid region of southeastern Spain as a case study. In this region the species is not abundant and at risk of local extinction due to climate change [18]. We built generalized linear models (using climate, and land use variables as predictors) to project the EVI-derived variables under the Inter-governmental Panel of Climate Change (IPCC) and land cover and land use change scenarios. Then, we incorporated the uncertainty derived from these models into future forecast by using the distribution of residuals. Finally, we discuss how the forecasted distribution based on our approach can guide policy-makers to review current policies and biodiversity conservation programs.

## Materials and methods

### Species model

The European badger is a medium-sized carnivore widely distributed across Europe. In the arid southeastern limits of its range (i.e., the Mediterranean drylands of Iberian Peninsula) the European badger prefers mosaic landscapes consisting of fruit orchards and natural vegetation, which provide shelter and food resources [19]. The potential effects of climate change on life-history traits such as population density, social organization or population growth have been highlighted for badger [20]. Although the species is currently considered of least concern (LC) in Spain [21], in the arid southeastern of Spain, badgers are not abundant and densities are usually below one badger/km^2^ [22]. Since the food resources exploited by the species directly or indirectly depend on the climate and human land use [23], it would be reasonable expect, under future change scenarios, that some territories currently occupied might be abandoned with the goal of finding areas with better conditions. Therefore, European badger is at potential risk of local extinction due to climate change [18], becoming an ideal study organism for the purpose of this study.

### Modeling approach

We first designed a set of SDMs at global scale (i.e., including the distribution range of the badger) using presence-only data and climate variables. We assembled them by a committee averaging method (see below a detailed description). Then, using the global model output to weight the pseudo-absences (PAs), we built four sets of SDMs at regional scale (i.e., in an arid region of southeastern Spain). Two of these sets included EVI-derived, climate and topographic variables (hereafter EVI-models). The other two sets included only climate and topographic variables (hereafter Climate-models). The pseudo-absences were just weighted in one set of EVI- and Climate-models, respectively [7]. Finally, we forecasted the potential spatial distribution of badger for the 2071–2099 period under IPCC scenarios [24] using the regional models with the best performance. Best performance was based on the area under the receiver operating characteristic curve (AUC). We note that to predict the future EVI-variables used in forecasting the potential spatial distribution, we built generalized linear models (GLMs) (using climate, and land cover and land use variables as predictors) and projected them under IPCC and land cover and land use change scenarios. We incorporated the uncertainty derived from GLMs into future forecast by using the distribution of residuals.

#### The global model

**Fig 1:**
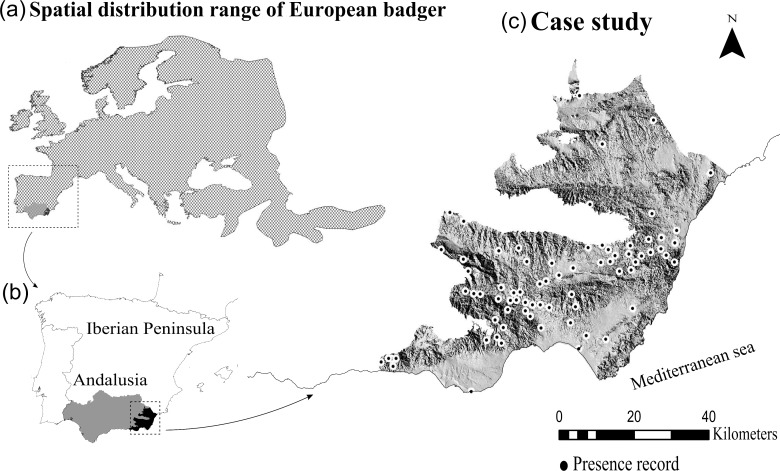
Spatial distribution of European badger. (a) Spatial distribution range of the European badger obtained from the IUCN map, (b) Regional administrative boundaries of Andalusia, (c) Case study in arid environments of southeastern Spain (7051 km^2^) defined using the Martonne aridity index and administrative boundaries of Andalusia; location of the 73 badger presence records used for the regional model. The digital elevation model showed was downloaded from a public database available in http://www.juntadeandalucia.es/institutodeestadisticaycartografia/prodCartografia/bc/mdt.htm

The spatial distribution range of badger was obtained from the Digital Distribution Maps of the IUCN Red List of Threatened Species (http://www.iucnredlist.org/) in vector format (Fig 1A). We extracted species occurrence from the Global Biodiversity Information Facility (http://data.gbif.org) at a minimum resolution of 2.5´ x 2.5´. We used 16279 records of occurrence after post-processing the data to remove those presences with unrealistic coordinates (e. g., sea, incomplete coordinates). From the 19 available bioclimatic variables at a 2.5´ spatial resolution of the WorldClim database (http://www.worldclim.org/), we selected seven with a Spearman rank-correlation lower than 0.6, i.e., annual mean temperature, mean diurnal range, temperature seasonality, temperature annual range, mean temperature of wettest quarter, annual precipitation and precipitation seasonality. Following to Gallien et al. [7], we modeled species distribution using six algorithms available in the “biomod2” package version 3.1–64 [25] in R (http://www.R-project.org/): a generalized additive model (GAM), a classification tree analysis (CTA), a multivariate adaptive regression splines (MARS), a boosted regression trees (BRT), an artificial neural networks (ANN) and a random forest (RF). We kept the default models options. For each algorithm, two PAs datasets (20000 each time) were randomly selected and four-fold cross-validations performed (48 models in total) by randomly selecting 70% of the presence locations to train the models, and the remainder 30% to evaluate them using the AUC [26]. Then, only those models obtaining an AUC score above 0.8 were used to build the committee averaging map. The committee averaging method is an ensemble forecasting method [27] by which the predicted probability maps of species habitat suitability are not averaged, but instead are transformed into binary maps (using for each model the threshold that maximizes both sensitivity and specificity). Thus, this method gives both a prediction and a measure of uncertainty. When the prediction is close to 0 or 1, it means that all models agree to predict 0 and 1 respectively (see [25] for details).

#### The regional model

For the regional model we selected an arid case study in the southeastern Iberian Peninsula (36°58’N, 2°29’W) (Fig 1C). This region is the most arid in all of Europe and presents the most extreme arid conditions in the specie range. We defined this area based on two criteria: (1) the Martonne aridity index (I_a_), only including values between 5 and 15 [28], (2) the administrative boundaries of the Andalusia (Fig 1B), because the methodologies employed to obtain environmental GIS cartography (used later to estimate environmental variables for the modeling process) are different across regional administrative boundaries (Spanish Autonomous communities).

The presence records for the badgers were obtained from published data [8] and personal databases from the authors (i. e., 184 occurrence registers). We reduced locally dense sampling by thinning the records to one per 5 x 5 km grid cell to avoid spatial autocorrelation problems. A total of 73 presence records were used in the modeling (Fig 1C). Presence samples were distributed across a wide gradient of altitude (0–1500 meters), temperature (annual minimum mean temperatures range: -1.6–15°C, annual maximum mean temperatures range 17–24.5°C), annual precipitation range (165–419 mm/year), and annual evapotranspiration range (343–1038 mm/year). Vegetation is mainly constituted by Mediterranean shrubland (e.g., *Pistacia lentiscus*, *Macrochola tenacissima*, *Anthyllis spp*.) with more xerophytic species in lower zones near to the coast (e.g., *Thymus spp*., *Salsola spp*.). Woodland is very scarce and is present inland and at higher altitude (e.g., *Pinus halepensis*). Extensive crops (e.g., fruit orchards) are associated with the main river courses where together with natural vegetation constitute the preferred habitats of the badger [8,19]. Intensive irrigated crops are dominant in areas further north (e.g., almond and arable crops).

We selected six variables associated to ecological requirements of the European badger and which have high predictive power in terms of habitat suitability [29,8]. Variables were related to climate (mean annual precipitation (*PREC*) and mean value of monthly maximum temperatures (*TMEDMAX*)), relief (mean slope (*SLOPE*)), and spatio-temporal patterns of primary production (EVI annual mean (*EVIMEAN*), intra-annual coefficient of variation of EVI (*EVIC*), and spatial standard deviation of EVI annual mean (*EVISTD*)). To avoid collinearity between predictors, we checked that Spearman rank-correlations were less than 0.85 [30]. The maximum Spearman correlation value obtained was -0.37, corresponding to *PREC* and *TMEDMAX*.

*PREC*, *TMEDMAX* (for the 1971 to 2000 period) and *SLOPE* were derived from spatial data layers of the Environmental Information Network of Andalusia (http://www.juntadeandalucia.es/medioambiente/site/web/rediam). *PREC* and *TMEDMAX* had a cell size (100 x 100 m), and *SLOPE* was calculated from a 20x20 m pixel digital elevation model of Andalusia. We resampled to the 231 x 231 m grid (pixel size of the MOD13Q1 EVI products, see below) using bilinear resampling, in Quantum GIS (QGIS; http://www.qgis.org) which is more realistic than nearest-neighbor interpolation [26]. This pixel resolution is suitable to capture the habitat preferences of the European badger at a local scale [8].

Our three functional descriptors of the spatiotemporal patterns of primary production were derived from satellite images captured by the MODIS sensor onboard the NASA TERRA satellite (http://www.modis.gsfc.nasa.gov/). We used the MOD13Q1 EVI product, which consists of 16-day maximum value composite images (23 per year) of the EVI at a 231 x 231 m pixel size. This product has atmospheric, radiometric and geometric corrections. We used EVI instead of NDVI because it is less influenced by soil background and saturation problems at high biomass levels [31]. We first used the Quality Assessment (QA band) information of this product to filter out those values affected by high content of aerosols, clouds, shadows, snow or water. Next, we calculated the mean seasonal EVI profile (average year) for the 2001–2013 period and derived the EVI annual mean (*EVIMEAN*) as the mean of the 23 images of the average year and the intra-annual coefficient of variation of EVI (*EVICV)* as the intra-annual standard deviation divided by *EVIMEAN*. The spatial standard deviation of *EVIMEAN* (*EVISTD*) was calculated in windows of 3 x 3 km (13x13 MOD13Q1 pixels) throughout the study area. The size of this window was determined based on the suggested 9 km^2^ home range of the European badger for low suitability habitats [19]. Horticultural greenhouses are intensively used in this area [32], and because the EVI values of greenhouses cannot be interpreted as vegetation greenness [31], we removed all grid cells containing greenhouses (5% of the study area) to avoid their influence of species distribution modeling.

To test the utility of functional descriptors variables in forecasting future spatial distribution of badger, we combined the six selected variables into two groups, with and without including EVI-derived variables. Then, we built four sets of regional SMDs, two of these sets included EVI-derived, climate and topographic variables and the other two only included climate and topographic variables. Using the global model output, the PAs were weighted only in one set of EVI- and Climate-models, respectively [7]. We followed the Gallien et al. approach [7] based on six statistical algorithms, i.e. GAM, CTA, GBM, RF, ANN and GLM. A PA datasets of 10000 points were randomly selected, followed by four cross-validation repetitions (70–30% on the presence locations for running and testing models respectively). To deal with the potential sample bias in the presence records (i.e., some sites are more likely to be surveyed than others), we followed the same sampling design for selecting PAs as for selecting presences [33]. In this manner, we restricted the choice of PAs inside the buffers of 5 km (size of plots used in the grid sampling by [8]) around any of the presence records. Ninety-six different models were finally run, and grouped into four sets: EVI-models with and without weighted PAs (24 models, respectively) and Climate-models with and without weighted PAs (24 models, respectively). Currently, an incompletely coverage of the environmental niche of species is recognized that reduces confidence of the forecasting distributions under climate change scenarios [4]. By focusing on a reduced area of the distribution range of badger (e.g., and arid southeast in the Iberian Peninsula), we are ignoring that the species may resist a broader range of environmental conditions, and thereby failing to take into account its ecological flexibility in responding to future climate conditions. To solve this point, we employed the approach supported by [7]. According to these authors, weighting the PAs in the regional model using the predictions from the global model enable: decrease the influence of (regional) false absences, validate the true (regional) presences, and let the regional climate, soil and land use refine the regional niche estimation. Thus, we used the global model projections applied to our case study to weight each PA. Where the global model showed a high level of agreement with the PA we attributed a high weight to the PA, and vice versa. The weights were given by the Eq (1):

$$\mathrm{Weight}\left(x\right)=\frac{1}{1+{\left(\frac{\mathrm{projG}\left(x\right)}{\mathrm{projG}\left(x\right)-1}\right)}^{2}}$$ (1)

where Weight(x) is the weight attributed to the PA x, and projG is the global model prediction at the location of x.

### Regional model evaluation and future forecasting

We evaluated the performance of regional models by AUC using the remainder of the presence records (i.e., 111) after selecting the samples used to build the models (see above). With the aim to obtain as reliable as possible future forecasting, we forecasted the badger spatial distribution for future climate scenarios in each set of models by using the best models based on AUC developed under current environmental conditions. A one-tailed Wilcoxon signed rank test was used to assess whether increases in overall AUC in the EVI-models relative to those in the Climate-models were statistically significant [34].

Two scenarios proposed by the IPCC were considered: A2 and B1. The A2 scenario assumes a continuously increasing global population, economic development that is primarily regionally oriented and focused on economic growth and technological changes that are more fragmented and slower than in other storylines. The B1 scenario assumes that economic structures rapidly change towards a service and information economy, and that resource-efficient technologies are introduced. The projected *PREC* and *TMEDMAX* (100 x 100 m cell size) variables for the 2071–2099 period were obtained from statistical downscaling methods (i.e., multiple linear regression) [35] of these IPCC scenarios available at the Environmental Information Network of Andalusia (see above). The simulated daily temporal series for the projected period were summarized by the annual mean with the aim of creating a representative year (see [36] for details). Then, we resampled to the 231 x 231 m grid using bilinear resampling. Although slope has been described as a relevant factor for sett digging, in low density areas of Mediterranean landscapes, setts were located almost everywhere [22]. Since slope seems not to be so limiting and was not available for future scenarios due to the complex drivers and predictors behind this process, we considered it static through time. The three projected functional descriptors derived from EVI (i.e., *EVIMEAN*, *EVICV* and *EVISTD*) were predicted by GLMs. Although precipitation and temperature are the main two climate factors that drive primary production of the biosphere, at the regional scale, the response of primary production to these climate drivers can vary both spatially and temporality, modulated by different vegetation types [37]. Therefore, *EVIMEAN* and *EVICV* were predicted by a GLM that used as explanatory variables the current *PREC* and *TMEDMAX* variables, land cover and land use and slope. Then, future *EVIMEAN* and *EVICV* were projected using the fitted models with future climate variables of each A2 and B1 scenario. In addition, we simulated three future scenarios of land cover-land use change: (1) irrigated crop scenario: the 5% and 10% of the natural vegetation and rainfed crop patches, respectively, converted into irrigated crop patches; (2) crop abandonment scenario: the 7.5% and 7.5% of rainfed and irrigated crop patches, respectively, converted into natural vegetation parches; (3) no land change scenario (see S1 Fig). These scenarios were based on the future projections obtained by Piquer-Rodríguez et al. [38] for the southeastern Iberian Peninsula and were simulated using the MOLUSCE plugin in QGIS (http://hub.qgis.org/projects/molusce). Finally, the future *EVISTD* variable was obtained by using a 3 x 3 km moving window on the projected *EVIMEAN* variable (as explained above).

In the linear regression, the predicted values of the response variable are estimated by the following Eq (2):

$$y_{i}=\beta_{0}+\beta_{1}*x_{i1}+\beta_{2}*x_{i2}+....+\beta_{j}*x_{ij}+\varepsilon_{i}$$ (2)

where y_*i*_ is the predicted value of variable response for the *i*th observation when the explanatory variable X_1_ equals x_*i*1_, X_2_ equals x_*i*2_, X_*j*_ equals x_*ij*_; β_0_ is the intercept; β_1_, β_2_ and β_*j*_ are the partial regression coefficients for the explanatory variables 1, 2 and *j*; Ɛ_*i*_ is unexplained error associated with the *i*th observation.

Errors are assumed to be normally distributed with expectation 0 and variance σ^2^. We exploited this assumption to incorporate the uncertainty derived from the projected EVI-derived variables into future forecasts. To do this, we generated 1000 projections of these variables by adding in each iteration the errors estimated from the GLMs. We obtained these errors using the distribution of residuals provided by the GLMs output (see S1 Table for details of GLMs).

Then, we assembled the future forecasts obtained using the projected climate and EVI-derived variables by the median value. All calculations were made using R software.

### Comparing current and future spatial distributions

We used a distance-based method [39] to compare the current and future spatial distributions yielded by the different regional models and scenarios. This method combines aspects of image analysis with distance-based statistical test to extract two types of information from an ensemble of SDM output maps: intensity and spatial difference.

Intensity index (Eq 3) measures the overall level of habitat suitability (based on the environmental variables used in the SDMs) for a species within an area extent but with the spatial information removed.

$$S_{i}=\overset{R}{\underset{r=1}{\sum }}\overset{C}{\underset{c=1}{\sum }}M_{i}\left[r,c\right]$$ (3)

where S_*i*_ is the intensity; M_*i*_ is the *i*th map in a set of SDM outputs; R is the number of rows indexing northing values in the map; C is the number of columns indexing easting values in the map.

Spatial differences between current and future distribution maps was tested by using the Hellinger distance (Eq 4) [40]. Wilson [39] found that this distance obtained the best results when SDMs output maps were spatially compared. Hellinger distance measures the dissimilarity between two probability density functions for continuous variables. Before to be used for comparing maps, it requires normalization by intensity, so that, the map has the properties of a bivariate probability distribution. Both measures (i.e., Hellinger distance and the corresponding difference in intensity) are largely independent and can be used in a scatterplot to show patterns of change in the distribution of habitat suitability [39,40].

$$H_{ij}={\left[0.5*\overset{R}{\underset{r=1}{\sum }}\overset{c}{\underset{c=1}{\sum }}{\left[\sqrt{\frac{M_{i}\left[r,c\right]}{S_{i}}}-\sqrt{\frac{M_{j}\left[r,c\right]}{S_{j}}}\right]}^{2}\right]}^{1/2}$$ (4)

where H_*ij*_ is the Hellinger distance between the M_*i*_ and M_*j*_ maps.

To check if both measures (i.e., intensity and Hellinger distance) yielded by Climate-model and EVI-models, respectively, were significantly different, we explored the probability distributions of the normalized pairwise differences for each measure by a bootstrap method (10.000 replicates) and then, we calculated which of these differences fell above the 95th percentile and below the 5th percentile.

## Results and discussion

### Model evaluations

All global models showed a good performance (AUC > 0.8) and therefore the 48 models were used in the ensemble modeling. However, when we selected the models at regional scale, we used different AUC values as thresholds with the aim to maximize the performances obtained. For the Climate-models, the AUC values were 0.55 and 0.6 for with and without weighted PAs models, respectively; in this form, 3 and 6 models were selected to forecast the badger spatial distribution into future scenarios. For the EVI-models, the AUC values used were 0.55 and 0.7 (with and without weighted PAs) and the number of models selected was 9 and 5, respectively.

**Fig 2:**
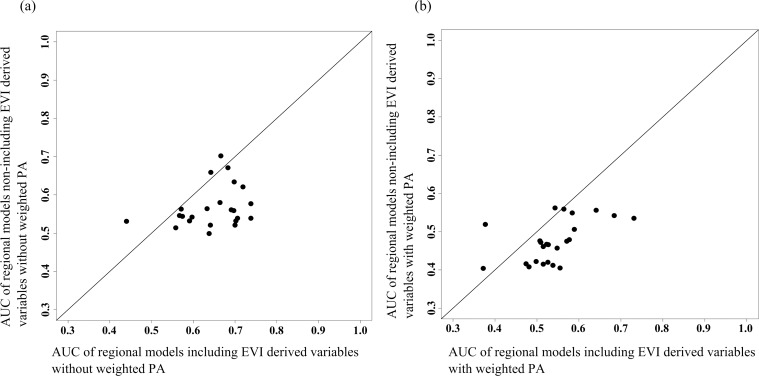
Comparison of model performance using the area under the threshold-independent receiver operating characteristic curve (AUC). (a) Regional EVI-model performance versus regional Climate-model, both without weighted PAs. (b) Regional EVI-model performance (AUC) versus regional Climate-model, both with weighted PAs. Values below the diagonal line mean a better performance for EVI-model, values above mean a better performance for Climate-model and values on the diagonal line mean an identical performance between the models compared.

EVI-models performed better than Climate-models (both without weighted PAs) (*W* = 453.5, *p*<0.0001) for the current conditions, although the AUC values accomplished were moderated (the maximum value reached was 0.74 into the EVI-models set) (Fig 2A). When we compared between models with weighted PAs, EVI-models were also better than Climate-models (*W* = 439, *p*<0.001), but the AUCs were still lower (Fig 2B). The comparison of the performance between models with and without weighted PAs but using the same type of variables, revealed that weighting the PAs did not significantly improved discrimination between areas where badger was present and where it was not recorded (PAs) (*W* = 473, *p*<0.0001; *W* = 506, *p*<0.00001).

### Comparing forecasted future distributions

**Fig 3:**
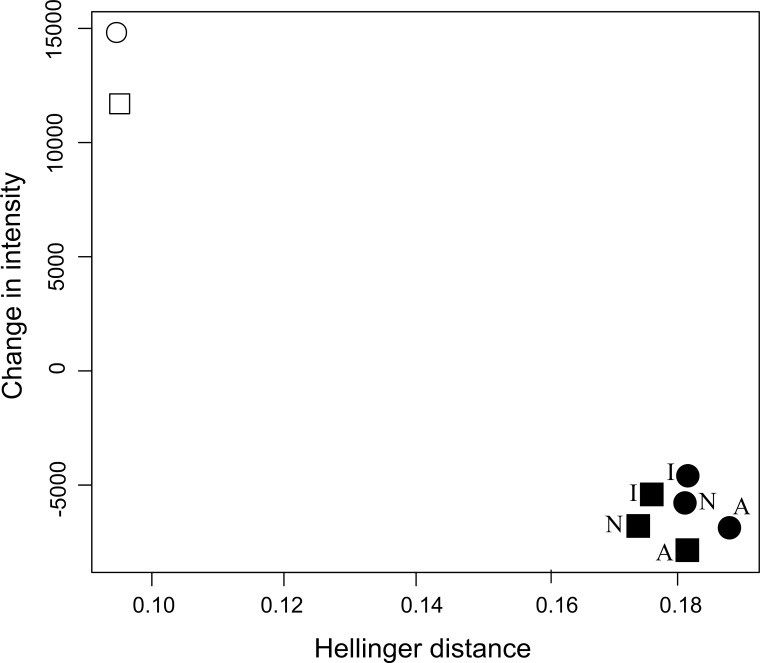
Changes in intensity (*Y* axis) and Hellinger distance (*X* axis) forecasted for the European badger in southeastern Iberian Peninsula under IPCC scenarios. Change in intensity (overall measure of habitat suitability) between the current and IPCC A2 (circles) and B1 (squares) for 2071–2099 maps is plotted against the corresponding Hellinger distance (representing spatial changes). Filled symbols represent EVI-models and open symbols Climate-models, both without weighted PAs. ***I***: *irrigated crop scenario;* ***A***: *crop abandonment scenario;* ***N***: *no change scenario*.

Because of the performance of models (i. e., Climate-models and EVI-models) with weighted PAs was lower than the models without weighted PAs, we only used the former to forecast future distribution. Badger was predicted to experience an increase in habitat suitability (positive change in intensity) and considerable spatial change when we just considered climate variables (Fig 3). A2 scenario forecasted the most favourable conditions for badger with these variables. However, when EVI-derived variables were incorporated into the distribution models, habitat suitability was predicted to decrease (negative change in intensity), and spatial pattern change was greater than without EVI-derived variables. The increase of spatial changes could be driven by LCLC change scenarios used to predict EVI variables. LCLU changes are considered to be a major driver of global environmental change and biodiversity loss [41]. For this reason, future forecasts that not include them, e.g. based only on climate variables, could harbour an important bias, particularly, at a regional scale where the landscape features, such as vegetation and land use, are key for the species [19,23]. Although the forecasts obtained from the different IPCC scenarios and land cover and land uses change were similar for EVI-models, the decrease of habitat suitability was more moderate with the increase of irrigated crop than crop abandonment. Finally, the forecasts predicted by Climate-models and EVI-models were significantly different at a 0.5 level of confidence (see Table A, Figs A and B in S1 File).

### EVI descriptors of ecosystem functioning to forecast species distributions

Our results suggest that future forecasts based only on climate variables should be interpreted with caution because the temporal dynamics and spatial patterns of ecosystem functioning may play an important role in determining the species distribution and the dynamics of its change. At a global scale, the bioclimate envelope approach (i.e., modeling the association between climate variables and species distribution) can provide a first approximation as to the potentially dramatic impact of climate change [5]. However, at a regional scale, factors that determine species distributions may vary [42] and therefore, the ecological limits predicted by climate may be misleading and the future forecasts obtained could underestimate or overestimate the potential changes in species distribution derived from climate change. In fact, we show how Climate- models and EVI-models forecasted opposed futures for the badger under both A2 and B1 scenarios in Mediterranean arid environments. Supporting this finding, Requena-Mullor et al. [8] showed that EVI descriptors of ecosystem functioning are useful to describe drivers of the spatial distribution of badger in these environments, in consequence, the future forecast derived from EVI-models may suggest more reliability.

At this point, a key question arises: how much more reliable are these future distribution forecasts?. Addressing this question goes beyond the goal of this study, however, our results show that EVI-derived variables could help to decrease the underlying ecological bias of the future forecasts based just on climate variables. In this regard, EVI-models forecasted a decreased of the habitat suitability for badger. Macdonald et al. [20] found that badger life history parameters (such as survival, fecundity or body-weight) are correlated with annual variability of both temperature and rainfall mediated by food supply. Therefore, climate trends might influence badgers population growth directly and through correlations with food availability [43]. Primary production is at the base of food webs, and therefore a decrease would translate into lower food availability for higher trophic levels. Supported by findings of Alcaraz-Segura et al. [12], we consider that EVI-derived variables could represent the interactions between climate and food availability for badger as descriptors of the temporal dynamics and spatial patterns of primary production. On a local scale, the composition of the badger diet is influenced primarily by human land use and management [23]. More specifically, in the southeastern Iberian Peninsula, badger diet depends on fruit orchards and other derived food resources (e.g., insects) [44]. EVI-derived variables have been suggested as proxies of the spatial and temporal variability of food resources for badgers in these environments [8]. Therefore, future forecasts based on both EVI-derived and climate variables would capture not only climate change but also correlations between climate and ecosystem functioning through the dynamic of primary production and land use change. According with this, our results showed that the decrease of habitat suitability with an increase of irrigated crops was less than with the abandonment crops.

The regional model performances were worse with weighted PAs. This result partially disagrees with findings by [7]. As we described above, at a regional scale, other factors than climate can play an important role in determining species distribution [42]. Although climate was not predicted as a limiting factor for the occurrence of badger in the arid southeastern of Iberian Peninsula by the global model (based only on climate variables, see S2 File), some authors have suggested that the species in these environments select rural landscapes consisting in a mosaic of fruit crops and orchards mixed with patches of semi-natural vegetation as a response to food shortage [19,8], which are not considered by the prediction of the global model. In consequence, the badger occurrence predicted by the global model in southeastern Iberian Peninsula was overestimated (see S2 File).

### Implications for biodiversity conservation policies

Since the validity of future forecast based only on climate variables is currently questioned [45], policies and biodiversity conservation programs supported by such information could have a biased vision and overestimate or underestimate the potential changes in the species distribution derived from climate change. The incorporation of ecosystem functional attributes derived from remote sensing in the modeling of future forecast may contribute to the improvement of detection of ecological responses under climate change scenarios. In addition, our approach would allow incorporating the uncertainty derived of these attributes when they are projected to future under both climate, and land use change scenarios. In consequence, the future forecast obtained would be more reliable and help policy-makers to review the existing policies and biodiversity conservation programs.

Regarding global biodiversity conservation efforts, we highlight that the use of variables related to ecosystem functional attributes derived from remote sensing data, has been shown to conduct cost-efficient monitoring schemes for biodiversity conservation and across a variety of ecosystems worldwide [46]. Our approach can be aligned with goals of the Group on Earth Observations Biodiversity Observation Network (GEO BON), which is seeking consensus for the key variables to improve the ever-increasing need for monitoring of biodiversity across space and time in a rapidly changing planet.

## Supporting information

S1 Fig Land cover-land use change maps. Simulated land cover and land use change scenarios.
(DOCX)

S1 Table GLMs for EVI-derived variables. Summary of the GLMs for *EVIMEAN* and *EVICV* variables.
(DOCX)

S1 File Differences of intensity and Hellinger distance. Normalized differences of intensity and Hellinger distance forecasted by Climate-models and EVI-models.
(DOCX)

S2 File Spatial distribution predicted for the European badger by the global model. Global model predictions and specificity of EVI-models.
(DOCX)
