# Supplementary figures and images for: Remote-sensing based approach to forecast habitat quality under climate change scenarios
Source: PLoS One. 2017 Mar 3;12(3):e0172107. doi: 10.1371/journal.pone.0172107 (PMC5336225; doi:10.1371/journal.pone.0172107)

**S1 Fig. Land cover-land use change maps.**


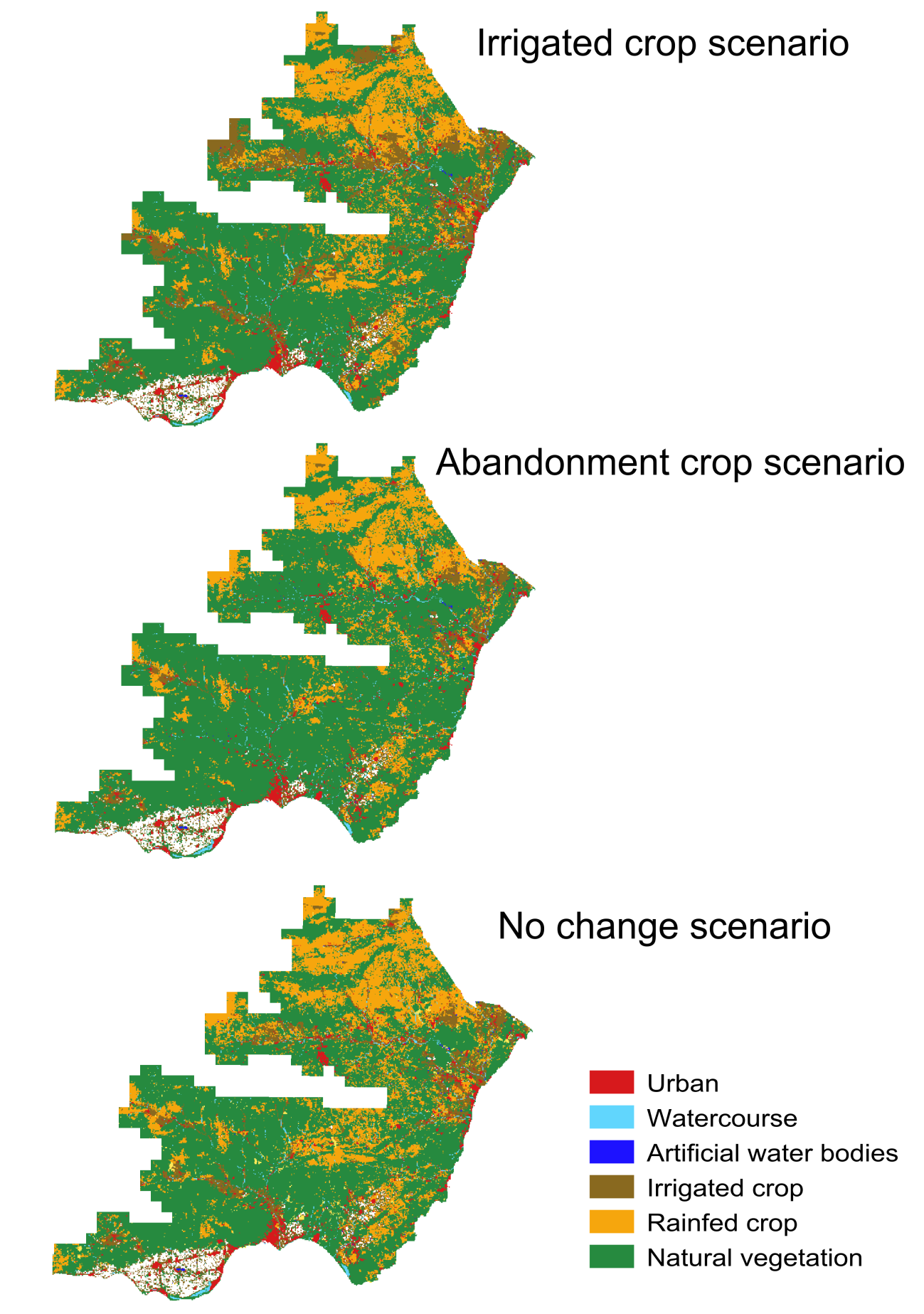


Legend. Simulated land cover and land use change scenarios.

Supplement: S1 Fig — Simulated land cover and land use change scenarios. (DOCX) [file pone.0172107.s001.docx]
