# Supplementary material for: Remote-sensing based approach to forecast habitat quality under climate change scenarios
Source: PLoS One. 2017 Mar 3;12(3):e0172107. doi: 10.1371/journal.pone.0172107 (PMC5336225; doi:10.1371/journal.pone.0172107)
Supplement: S1 Table — Summary of the GLMs for EVIMEAN and EVICV variables. (DOCX) [file pone.0172107.s002.docx]

**S1 Table. GLMs for EVI-derived variables.**

Table S1. Summary of the GLMs for *EVIMEAN* and *EVICV* variables.

| (a) *EVIMEAN* linear model | |
| --- | --- |
| Null deviance | 142.2 |
| Residual deviance | 113.12 |
| Explained deviance | 29.08 |
| Explained percentage | 20.45% |
| Residual variance (used to predict *EVIMEAN* values) | 0.00106 |

| (b) *EVICV* linear model | |
| --- | --- |
| Null deviance | 426.06 |
| Residual deviance | 369.85 |
| Explained deviance | 56.21 |
| Explained percentage | 13.19% |
| Residual variance (used to predict *EVICV* values) | 0.0034 |
