# Supplementary material for: Remote-sensing based approach to forecast habitat quality under climate change scenarios
Source: PLoS One. 2017 Mar 3;12(3):e0172107. doi: 10.1371/journal.pone.0172107 (PMC5336225; doi:10.1371/journal.pone.0172107)

**S1 File. Differences of intensity and Hellinger distance.**

Table A. Normalized differences of intensity and Hellinger distance forecasted by Climate-models and EVI-models.

| Scenarios | Intensity difference | Hellinger distance difference |
| --- | --- | --- |
| CA2 - CB1 | 0.34281112 | -0.011985743 |
| EVIB1N - CB1 | -2.031516915 | 1.976079769 |
| EVIB1A - CB1 | -2.152797379 | 2.171060497 |
| EVIB1I - CB1 | -1.873409604 | 2.029863346 |
| EVIA2N - CB1 | -1.915897713 | 2.16176456 |
| EVIA2A - CB1 | -2.04059155 | 2.338476916 |
| EVIA2I - CB1 | -1.779953564 | 2.173087795 |
| EVIB1N - CA2 | -2.374328035 | 1.988065512 |
| EVIB1A - CA2 | -2.495608499 | 2.18304624 |
| EVIB1I - CA2 | -2.216220723 | 2.041849089 |
| EVIA2N - CA2 | -2.258708833 | 2.173750303 |
| EVIA2A - CA2 | -2.38340267 | 2.350462659 |
| EVIA2I - CA2 | -2.122764683 | 2.185073538 |
| EVIB1A - EVIB1N | -0.121280464 | 0.194980728 |
| EVIB1I - EVIB1N | 0.158107311 | 0.053783577 |
| EVIA2N - EVIB1N | 0.115619202 | 0.185684791 |
| EVIA2A - EVIB1N | -0.009074635 | 0.362397147 |
| EVIA2I - EVIB1N | 0.251563352 | 0.197008026 |
| EVIB1I - EVIB1A | 0.279387776 | -0.141197151 |
| EVIA2N - EVIB1A | 0.236899666 | -0.009295937 |
| EVIA2A - EVIB1A | 0.11220583 | 0.167416419 |
| EVIA2I - EVIB1A | 0.372843816 | 0.002027298 |
| EVIA2N - EVIB1I | -0.042488109 | 0.131901214 |
| EVIA2A - EVIB1I | -0.167181946 | 0.30861357 |
| EVIA2I - EVIB1I | 0.09345604 | 0.143224449 |
| EVIA2A - EVIA2N | -0.124693837 | 0.176712356 |
| EVIA2I - EVIA2N | 0.135944149 | 0.011323235 |
| EVIA2I - EVIA2A | 0.260637986 | -0.165389121 |

***C****: Climate-models;* ***EVI****: EVI-models;* ***B1****: IPCC scenario B1;* ***A2****: IPCC scenario A2;* ***N****: no change scenario;* ***A****: crop abandonment scenario;* ***I****: irrigated crop scenario*.

Fig. A. Distribution of intensity differences.


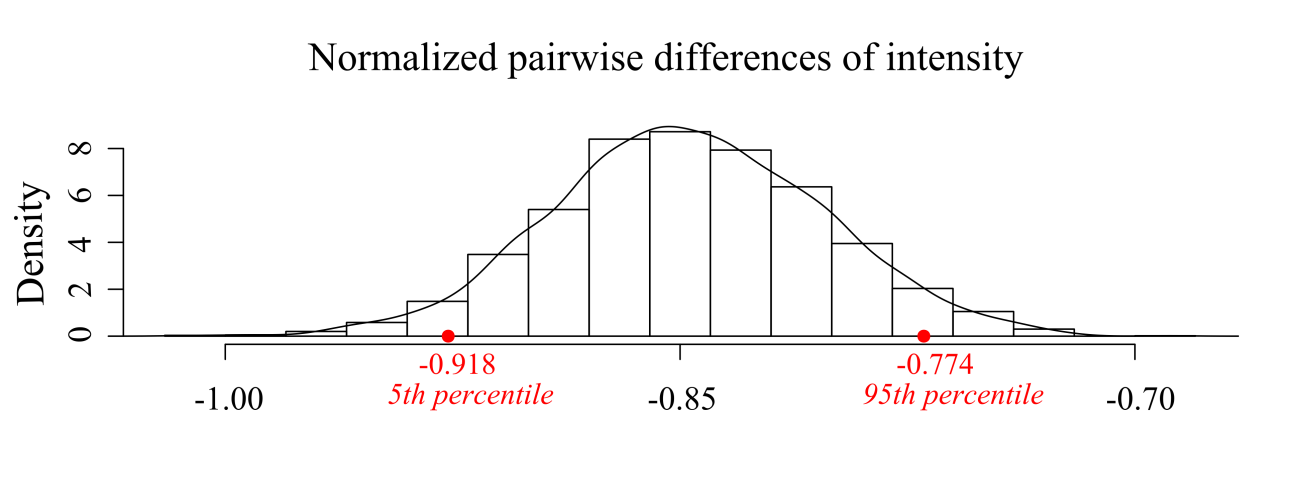


Fig. B. Distribution of Hellinger distance differences.


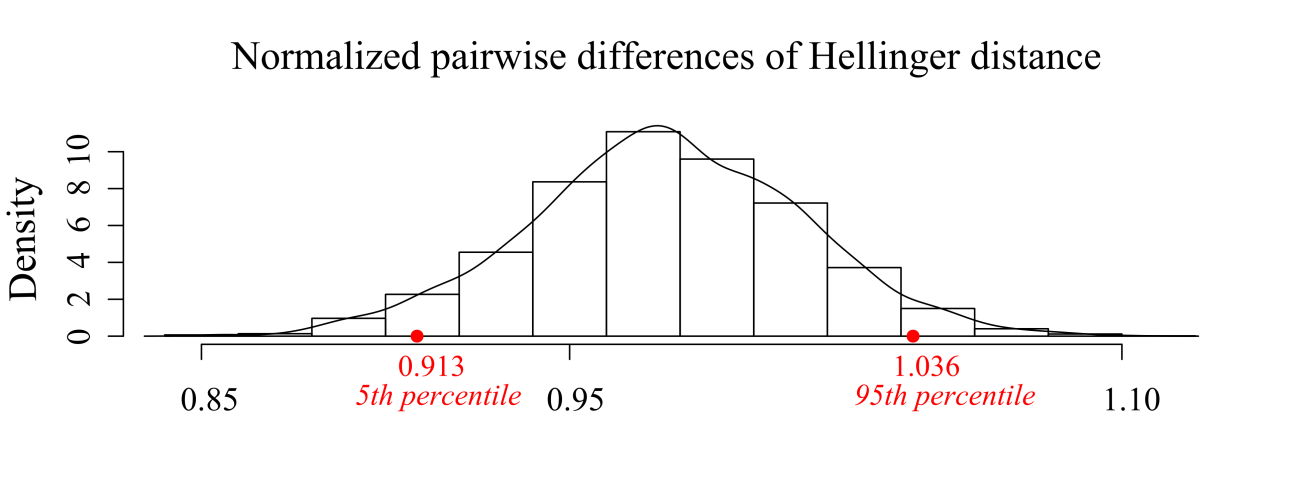

Supplement: S1 File — Normalized differences of intensity and Hellinger distance forecasted by Climate-models and EVI-models. (DOCX) [file pone.0172107.s003.docx]
