# Supplementary material for: Remote-sensing based approach to forecast habitat quality under climate change scenarios
Source: PLoS One. 2017 Mar 3;12(3):e0172107. doi: 10.1371/journal.pone.0172107 (PMC5336225; doi:10.1371/journal.pone.0172107)

**S2 File. Spatial distribution predicted for the European badger by the global model.**

Global model predicted a high percentage of agreement on badger presence between the various algorithms in the arid southeastern Spain (Fig A).

Specificity (i.e., proportion of absences correctly predicted) in EVI-models with pseudo-absences (PAs) weighted was better than EVI-models without weighted Pas (see Fig A) because badger occurrence predicted by Global model was overestimated.

Specificity was estimated using for each model the threshold that maximizes both sensitivity and specificity.

Fig A. Global model predictions and specificity of EVI-models.


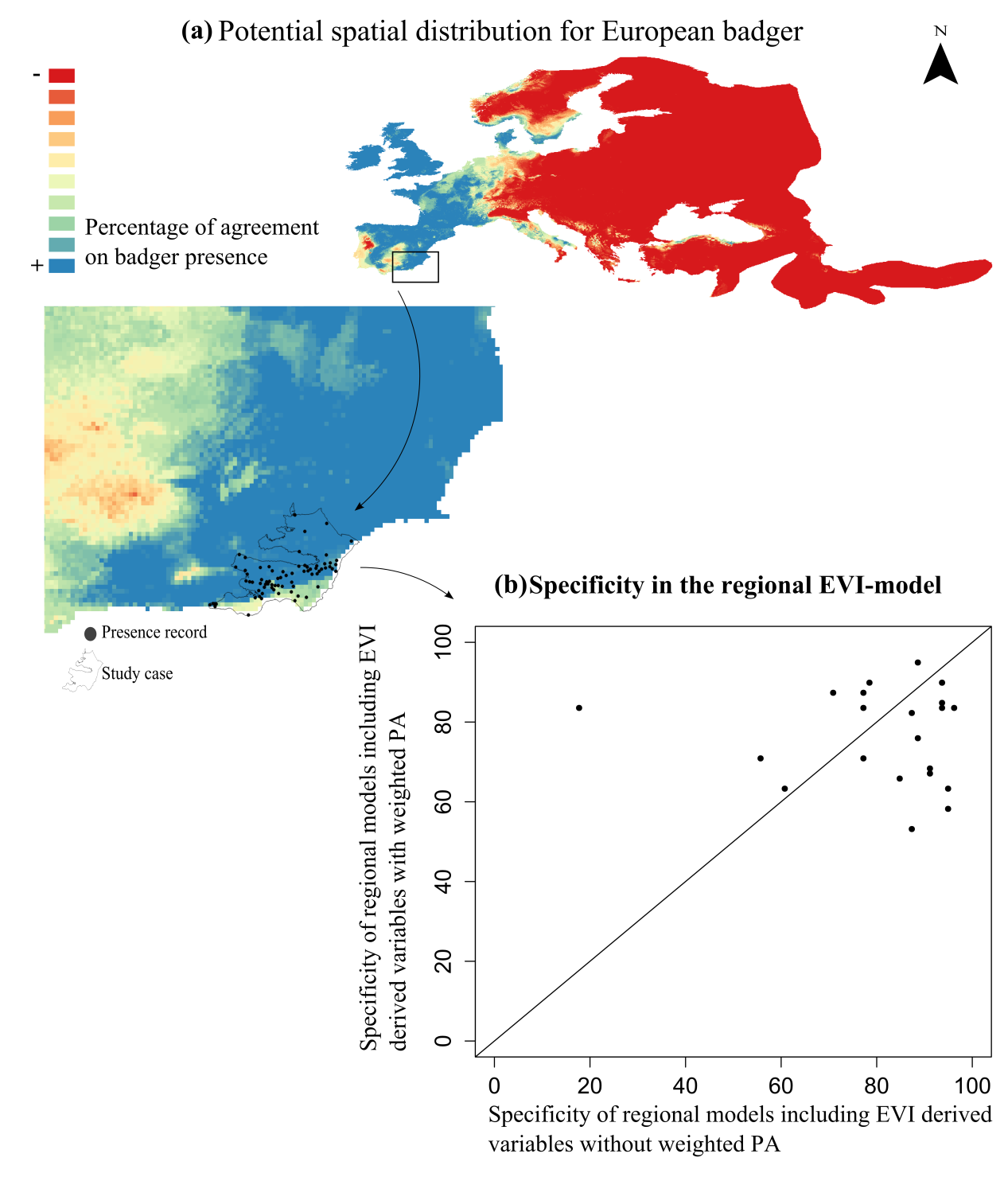

Supplement: S2 File — Global model predictions and specificity of EVI-models. (DOCX) [file pone.0172107.s004.docx]
